# Supplementary material for: Enhanced cleavage of genomic CCR5 using CasX2Max
Source: RNA Biol. 2025 Oct 25;22(1):1–18. doi: 10.1080/15476286.2025.2577449 (PMC12562720; doi:10.1080/15476286.2025.2577449)
Supplement: Supplemental Material [file KRNB_A_2577449_SM6403.zip › Supplemental files - updated version/CasX2MAX cleavage of CCR5 Supplemental text.docx]

**Supplemental Figure S1.**

**
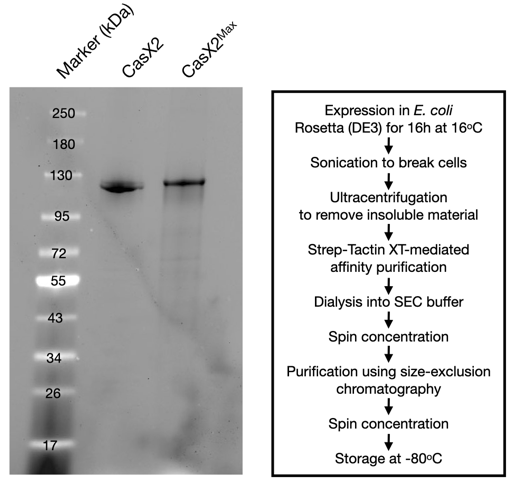
**

**Supplemental Figure S1. SDS-PAGE analysis of purified CasX2 and CasX2^Max^.** **A.** Stain-Free gel showing purified recombinant CasX2 and CasX2^Max^, each bearing an N-terminal SV40 nuclear localization signal (NLS) and C-terminal nucleoplasmin NLS, 3× hemagglutinin (HA) epitope tag, and Twin-Strep tag. Both proteins migrate near their expected molecular weight (~123 kDa) with no detectable degradation products or significant contaminants. Molecular weight markers (kDa) are shown at left. **B**. Workflow for CasX2 and CasX2^Max^ protein expression and purification.

CasX2 and CasX2^Max^ were expressed in *E. coli* Rosetta (DE3) cells for 16 h at 16^o^C following IPTG induction in Terrific Broth. Cells were lysed by sonication after nuclease treatment, and lysates were clarified by ultracentrifugation at 50,000 *× g*. Soluble protein was purified via Strep-Tactin XT affinity chromatography, dialyzed into SEC buffer, and concentrated using 50 kDa MWCO spin concentrators. Further purification was performed by size-exclusion chromatography on a Superdex 200 column. Final CasX2 preparations were concentrated, snap-frozen in liquid nitrogen, and stored at −80^o^C.

**Supplemental Figure S2.**

**
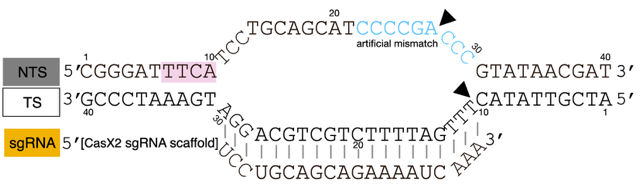
**

**Supplemental Figure S2. Reference sequence and base numbering used for structural models and interaction mapping.** Schematic of the CasX2 target duplex and guide RNA used throughout this study, adapted from the protospacer sequence resolved in cryo-EM structures (e.g., PDB: 7WAY). The non-target strand (NTS) and target strand (TS) are shown with base positions numbered 1–40 for consistency across all structural figures. The 5′ end of the sgRNA spacer hybridizes to the TS and extends toward the sgRNA scaffold (not shown). The PAM sequence (TTCA) is highlighted in pink. Transversion mutations in the NTS (blue) introduce a local mismatch that destabilizes the duplex and promotes R-loop formation for cryo-EM analysis. Triangles (black) indicate the resolved cleavage sites on the NTS and TS observed in structural states I and II. Color blocks to the left denote the standard strand color scheme used throughout the manuscript: NTS (dark gray), TS (white), and sgRNA (gold).

**Supplemental Figure S3.**

**
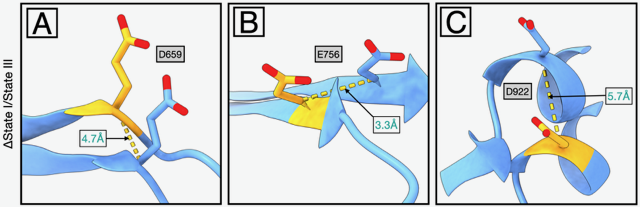
**

**Supplemental Figure S3. Positional displacement of CasX2 catalytic residues between active and inactive conformations.** The positions of the RuvC catalytic triad residues in CasX2 are shown in the active conformation (state I, blue) and the catalytically inactive conformation (state III, yellow). Superimposed backbone models highlight α-carbon shifts for (**A**) D659 (4.7 Å), (**B**) E756 (3.3 Å), and (**C**) D922 (5.7 Å), reflecting localized remodeling of the RuvC domain during the transition to state III.

**Supplemental Figure S4.**


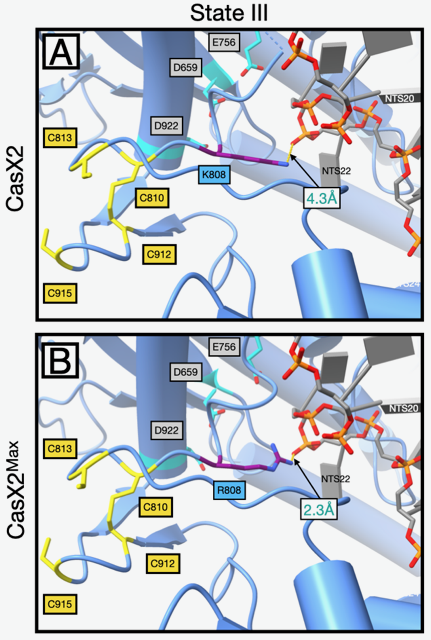


**Supplemental Figure S4. K808R substitution restores NTS phosphate contact in the inactive state III conformation. A.** In native CasX2, state III rearrangements disrupt the zinc ribbon and reposition K808, increasing its distance to the NTS phosphate at position 22 to 4.3 Å. **B.** In CasX2^Max^, the K808R substitution reduces this distance to 2.3 Å, maintaining contact with the NTS backbone despite the same state III conformation. Residues and strands are colored as follows: K808 or R808 side chains, purple; zinc-coordinating cysteines of the zinc ribbon motif (C810, C813, C912, C915), yellow; RuvC catalytic residues D659, E756, and D922, cyan; DNA non-target strand (NTS), dark gray, with phosphodiester backbone atoms shown as phosphorous (orange) and non-bridging oxygens (red).

**Supplementary Table 1 (Table S1):** List of gRNA spacer sequences used.

**Supplementary Table 2 (Table S2):** List of sgRNA scaffold sequences used.

**Supplementary Table 3 (Table S3):** List of plasmids used with complete plasmid sequence.

**Supplementary Table 4 (Table S4):** List of oligonucleotides used for cloning of gRNAs.

**Supplementary Table 5 (Table S5):** List of PCR primers used in Figures 1, 3, 4, 5 and 6.

**Supplementary Table 6 (Table S6):** List of PCR amplification conditions used in Figures 1, 3, 4, 5 and 6.
